# A Crowdsourced Megastudy of 12 Digital Single-Session Interventions for Depression in American Adults

**DOI:** 10.21203/rs.3.rs-7236847/v1

**Published:** 2025-08-25

**Authors:** Benjamin Kaveladze, Jan Voelkel, Michael Stagnaro, Mingjing Huang, Amanda Smock, Erin Sullivan, Yao Xu, Madison McCall, Juan Pablo Zapata, Syed Ishtiaque, Ananya Bhattacharjee, Iva Georgieva, Rosa Hernandez-Ramos, Kenneth Huber, Julia Jennings, Arianna Kirk, Rachel Kornfield, Robert Knowles, Monika Lind, Michelle Liu, Michael Liut, Alex Mariakakis, Ali Mattu, Adam McGuire, Jonah Meyerhoff, Alissa Mrazek, Michael Mrazek, David Mohr, Robert Morris, Christopher Mosunic, Hana Nip, Allen Olson-Urtecho, Jessa Podell, Dustyn Ransom, Shireen Rizvi, Matthew Southward, Sarah Elizabeth Stoeckl, Madison Taylor, Amy Texter, Angelique Trotter, Calvin Tower, Joseph Williams, Elijah Woodson, Katherine Wislocki, John Protzko, Lorenzo Lorenzo-Luaces, Stephen Schueller, Matthew Nock, Jessica Schleider

**Affiliations:** University of California, Irvine, USA; Cornell University; Yale University; Northwestern University; Northwestern University; Central Connecticut State University; Stanford University; University of North Carolina at Chapel Hill; Northwestern University; University of Toronto; University of Toronto; Bulgarian Academy of Sciences; University of California, Irvine; Calm Health; University of Texas at Austin; Visionari Media; Northwestern University; Koko; University of California, Irvine; Loyola University Chicago; University of Toronto Mississauga; University of Toronto; Mind Stuff LLC; The University of Texas at Tyler; Northwestern University Feinberg School of Medicine; The University of Texas at Austin; The University of Texas at Austin; Northwestern University; Koko; Calm Health; University of California, Irvine; Studio Bahia; The University of Texas at Austin; The University of Texas at Austin; Albert Einstein College of Medicine; The Ohio State University; University of California, Irvine; University of California, Irvine; University of Colorado Boulder; The University of Texas at Austin; The University of Texas at Austin; University of Toronto; The University of Texas at Austin; University of California, Irvine; Central Connecticut State University; Indiana University Bloomington; University of California Irvine; Harvard University; Northwestern University

## Abstract

Digital self-guided single-session interventions (SSIs) provide a structured psychological intervention within one interaction. We crowdsourced 66 diverse 10-minute SSIs for depression and selected 11 for testing in a pre-registered “megastudy” (ClinicalTrials.gov ID: NCT06856668). American adults (N = 7,505) experiencing elevated depressive symptoms were recruited online and randomly assigned to one of the 11 crowdsourced SSIs, a previously-validated active comparison SSI, or a control without intervention content. Nearly all SSIs boosted agency and hope for improvement immediately after completion (ds ≤ 0.37). However, only two significantly reduced depression at four-week follow-up (ds = 0.14 and 0.15). Unexpectedly, some SSIs may have decreased readiness to change at four weeks (ds ≤ 0.14). The most successful SSIs provided focused, engaging, and actionable guidance on a skill that directly addressed users’ struggles. Future work should aim to leverage SSIs’ short-term gains to promote longer-term behavior change or service engagement.

## Introduction

Depression is a major global health concern and a profound burden for the roughly 280 million people it affects each year (Marcus et al., 2012; World Health Organization, 2022). Evidence-based psychological interventions like psychotherapy can empower people with the skills and confidence to prevent and treat their depression (Cuijpers, 2019). However, barriers like cost, lack of providers, and stigma make in-person psychological interventions difficult to access for most (Kazdin & Blase, 2011). Digital mental health interventions are more scalable than in-person options, offering users on-demand care at low or no cost via computers and mobile devices (Ramos et al., 2024). Yet, these tools have had limited impact due to struggles reaching broad populations and engaging users effectively enough to produce lasting change (Jardine et al., 2024; Krukowski et al., 2024; Yardley et al., 2016).

Single-session interventions (SSIs) are structured programs designed to spark meaningful gains in mental health within a single interaction (Schleider et al., 2020). SSIs sidestep key barriers to both in-person and digital support, most notably the need for long-term engagement. SSIs can be delivered in various formats: in-person or online, self-guided or human-supported, and targeting outcomes in the SSI user or in others (e.g., equipping parents with skills to better support their children). In this paper, we hereafter use the term “SSI” to refer to SSIs that are digital, self-guided, and aim to improve the SSI user’s own mental health.

SSIs’ lightweight nature makes them ideally suited for broad dissemination (Schleider & Beidas, 2022). SSIs have already been implemented across diverse populations and settings, including psychotherapy waitlists, social media, schools, and war zones (Schleider et al., 2025). In many cases, these deployments helped people who were unlikely to have received a reliable intervention otherwise (Dobias et al., 2022).

### SSI Efficacy

SSIs can improve a range of mental health outcomes with small average effects (Schleider et al., 2025). However, the evidence specifically regarding SSIs for depression remains mixed. A meta-analysis of 15 randomized controlled trials (RCTs) of SSIs found a small overall effect on youth depressive symptoms (*g* = −0.12, 95% CI: −0.23, −0.01), with substantial heterogeneity (*I*^2^ = 58%) and a wide prediction interval (−0.44, 0.20; Ball et al., 2024). However, other well-powered trials of SSIs with adult samples have not found significant effects on depression (Lorenzo-Luaces & Howard, 2023; Lorenzo-Luaces et al., 2024) or related outcomes like anxiety (Mullarkey et al., 2022). Key questions remain about the populations for which SSIs are suitable (e.g., adults versus adolescents) and how different SSIs compare in meeting specific populations’ needs.

SSIs are not a replacement for psychotherapy nor sufficient for durable behavior change. Rather, they are a flexible adjunct to existing, often inaccessible, forms of mental health treatment. While SSIs’ average effects are small at an individual level, if implemented at scale and in settings in need of on-demand support, they could have a profound public mental health impact (Dodge et al., 2024, Prentice & Miller, 1992).

### SSI Design

Current evidence-based SSIs typically focus on a core concept (e.g., “taking positive action can change your brain for the better”), reinforced through interactive exercises (Manvelian et al., 2025). Their durations vary, usually between 5 and 30 minutes. These SSIs aim to introduce evidence-based skills and immediately boost hope and agency in ways that might translate to lasting change (Schleider, Abel, et al., 2019).

Some best practices for SSI design have been proposed, building on evidence from longer digital mental health interventions, mindset interventions (Yeager & Dweck, 2012), and bibliotherapy self-help books (Gualano et al., 2017). For example, the *B.E.S.T.T. elements* of SSIs for youth mental health recommends **b**rain science to normalize concepts and enhance credibility, **e**mpowering users to step into a “helper” or “expert” role, “**s**aying-is-believing” exercises to solidify learning, **t**estimonials and evidence from valued others about SSIs’ benefits, and **t**argeting specific outcomes (Manvelian et al., 2025; Schleider, Dobias, Sung, Mumper, et al., 2020).

SSIs aligning with these design principles provide solution-focused guidance in a clear and acceptable format (Manvelian et al., 2025; Schleider, Dobias, Sung, Mumper, et al., 2020). Yet, these approaches may not be optimal for all problems and populations. Exploring novel design directions (i.e., intervention strategies and stylistic elements) could advance SSIs toward greater efficacy and broader appeal.

### Megastudies

An efficient way to explore the potential of a range of SSIs is to conduct a “megastudy.” Megastudies experimentally compare many interventions simultaneously using common outcomes, control conditions, and sampling population (Voelkel et al., 2024). This “apples to apples” comparison enables faster, clearer insights than aggregating results across disparate trials. By showing which approaches work and which do not, megastudies can help developers and researchers to direct their efforts toward better interventions, accelerating fieldwide progress (Milkman et al., 2021).

Megastudies have been used in several contexts. For example, one field megastudy that randomly assigned 61,293 members of a fitness chain to one of 53 interventions encouraging gym attendance found that offering small rewards for returning to the gym after a missed workout increased weekly visits by up to 27% (Milkman et al., 2021). Another field megastudy (*n* = 689,693) showed that a text message reminder sent twice prior to a primary care visit boosted vaccination rates by 5% (Milkman et al., 2022). A third megastudy tested 25 brief online interventions targeting anti-democratic attitudes and partisan animosity in a sample of 32,059 Americans, finding the most successful interventions highlighted commonalities between likeable individuals with different political beliefs (Voelkel et al., 2024).

The first step in organizing a megastudy is to search for interventions to include. Popular interventions and those with robust evidence are useful starting points for this search, but they could overshadow innovative contributions from underrecognized teams without the means for rigorous testing. Crowdsourcing, in which many people are invited to contribute an intervention for testing, can expand megastudies’ scopes to include these potential hidden gems. For example, the political megastudy noted above crowdsourced 252 submissions from global teams of both academics and real-world practitioners (Voelkel et al., 2024).

### The Current Study

Given SSIs’ capacity for scale, finding innovative ways to increase their efficacy and appeal may bring them closer to fulfilling their promise of global impact. We conducted a pre-registered crowdsourced megastudy to identify and evaluate a diverse set of SSIs for depression in American adults reached via online participant recruitment platforms. We compared SSIs’ efficacy and appeal against a previously-validated evidence-based active comparator SSI as well as an attention-matched control providing information unrelated to mental health (Schleider et al., 2022). Analyses examined which SSIs significantly reduced depression and improved related outcomes, and which features distinguished the most and least helpful SSIs.

## Results

Data collection occurred from March to June 2025. After exclusions, of the 7,505 participants randomized, 7,338 finished the first session and 5,824 finished the four-week follow-up (77.6% of those randomized). The sample’s mean age was 36.6 years (SD = 11.8), 59% were women and 36% were men, 74% were White and 14% were Black, and 60% had received some form of mental health support in the past month. The trial CONSORT diagram is available online (https://osf.io/b7c86), as are participants’ baseline demographic characteristics (https://osf.io/jzbkq).

The height of the colored area shows the number of participants who reported the score shown on the horizontal axis at baseline. The green, vertical, dot-dash line shows the median value for each outcome. For depressive symptoms, scores from 5 to 9 indicate mild depression, 10 to 14 indicate moderate depression, 15 to 19 indicate moderately severe depression, and 20 to 27 indicate severe depression.

### Preliminary Analyses

Among participants in the attention control condition, depression (PHQ-9) decreased from baseline to week four (*b* = −2.99; *t*(1355) = −19.76; Cohen’s *d* = −0.58; 95% CI −0.64, −0.52; *p* = 1.46 * 10^−76^). Among those who completed both timepoints, depression at baseline correlated with depression at week four at *r*(1502) = 0.48; 95% CI 0.43, 0.52; *p* = 2.42 * 10^−69^. 2.0% of participants dropped out of the study while completing the SSIs or control, and the dropout rate differed across conditions (*X*^2^[12] = 71.65, *p* = 1.57 * 10^−10^). Dropout was < 2.6% in all conditions but Mindful Acceptance, which had 6.2%. The attrition rate at four-week follow-up was 21.0% and did not differ across conditions (*p* = .18). 99.9% of participants passed the attention check item, and 99.8% spent at least five minutes on the main survey (median duration 20 minutes).

### Change in Outcomes across Conditions at Immediate Post-test

**Figure 2:**
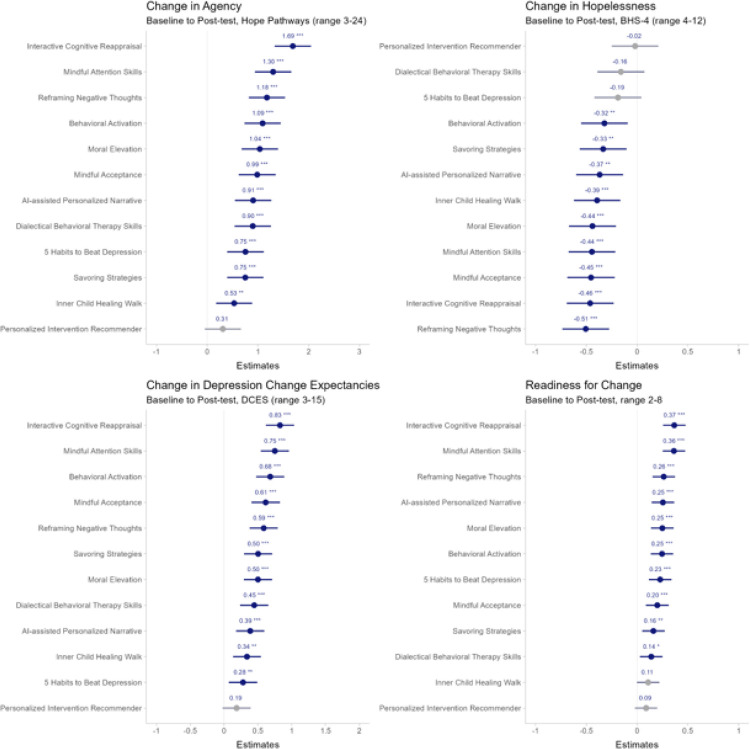
Intervention Effects at Immediate Post-test in a Megastudy (*N* = 7,505) of 12 Single-Session Interventions for Depression

The 12 SSIs (available at https://osf.io/nqdte) were each compared to the control, a 10-minute educational program unrelated to mental health, in their effects on psychological outcomes. Immediately after completing the treatment, participants assigned to 11 of the 12 SSIs had greater improvements in agency than those assigned to the control (*d*s: 0.12 – 0.37; *p*s: .003 – 4.02*10^−20^); those assigned to nine of the SSIs had greater reductions in hopelessness (*d*s: −0.11 – −0.18; *p*s: .01 – 1.65*10^−5^); those assigned to 11 of the SSIs had greater gains in depression change expectancies (*d*s: 0.12 – 0.35; *p*s: .01 – 9.93*10^−15^); and those assigned to 11 of the SSIs had greater increases in readiness to change (*d*s: 0.11 – 0.28; *p*s: .01 – 1.11*10^−10^); refer to Fig. 2.

In a pre-registered secondary analysis, compared to the active comparator *Behavioral Activation* SSI, those assigned to two SSIs (*Personalized Intervention Recommender* [*d* = −0.17; *p* = 4.26*10^−4^] and *Inner Child Healing Walk* [*d* = −0.12; *p* = .01]) had smaller increases in agency at post-test, while one SSI had a larger increase (*Interactive Cognitive Reappraisal*, *d* = 0.13; *p* = .01). Those assigned to one SSI had a smaller reduction in hopelessness at post-test (*Personalized Intervention Recommender* [*d* = 0.10, *p* = .04]). Those assigned to four SSIs had smaller increases in depression change expectancies at post-test (*d*s = −0.12 – −0.21, *p*s = 0.02 – 1.24*10^−4^). Lastly, those assigned to two SSIs (*Personalized Intervention Recommender* [*d* = −0.12, *p* = .02] and *Inner Child Healing Walk* [*d* = −0.11, *p* = .047]) had smaller increases in readiness to change.

### Change in Outcomes across Conditions at Four-week Follow-up

**Figure 3:**
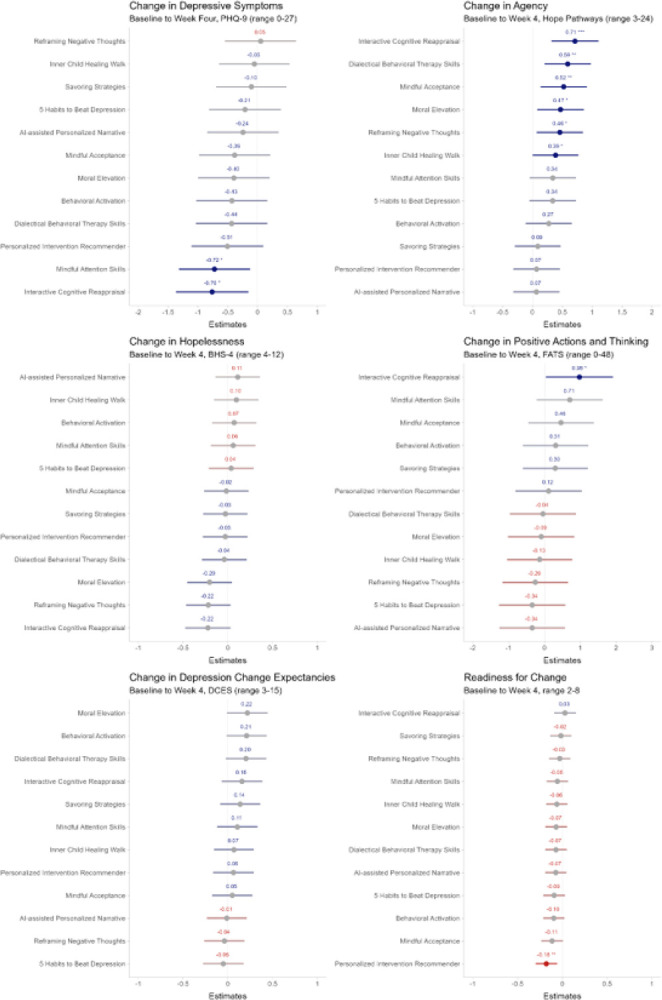
Intervention Effects at Four-week Follow-up in a Trial (*N*= 7,505) of 12 Single-Session Interventions for Depression

**Figure 4:**
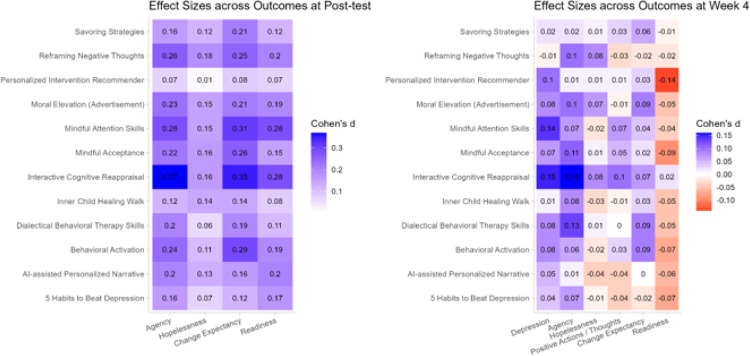
Effect Sizes by Outcome and Condition at Post-test and Week Four in a Trial (N = 7,505) of 12 Single-Session Interventions for Depression

**Figure 5:**
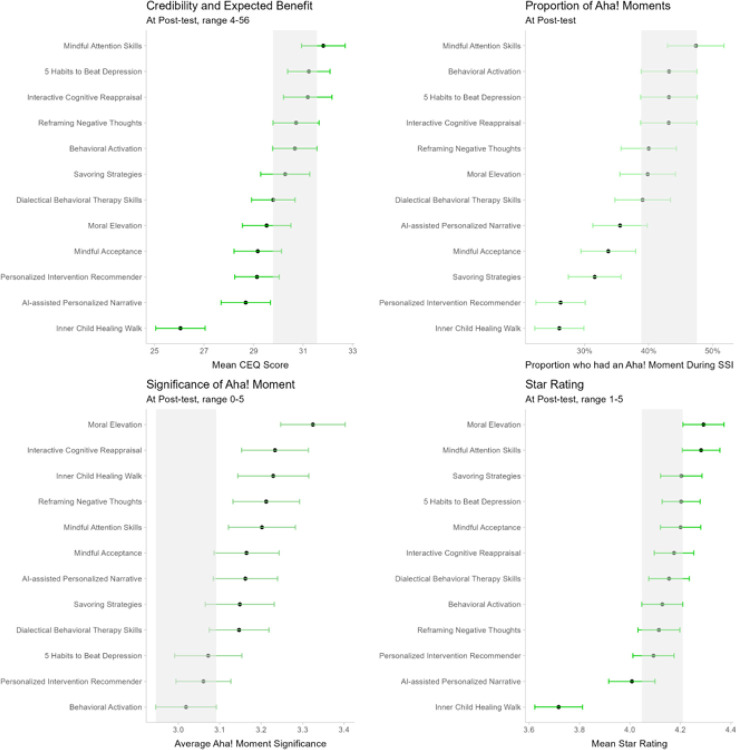
Acceptability Outcomes in a Trial (*N* = 7,505) of 12 Single-Session Interventions for Depression

At four-week follow-up, compared to those assigned to the control, participants assigned to *Interactive Cognitive Reappraisal* (*b* = −0.76; *t*(6813) = −2.46 *d* = −0.15; 95% CI −0.26, −0.03; *p* = .01) and *Mindful Attention Skills* (*b* = −0.72; *t*(6704) = −2.37; *d* = −0.14; 95% CI −0.25, −0.02; *p* = .02) had greater reductions in depression. Participants assigned to six of the SSIs had greater increases in agency (*d*s .08 – 0.16, *p*s .048 – 3.96*10^−4^), and those assigned to *Interactive Cognitive Reappraisal* had greater gains in positive actions and thinking (*b* = 0.98; *t*(6407) = 2.04; *d* = 0.10; 95% CI 0.00, 0.20; *p* = .04). Yet, participants assigned to *Personalized Intervention Recommender* showed smaller increases in readiness to change (*b* = −0.18; *t*(13595) = −2.90; *d* = −0.14; 95% CI −0.04, −0.23; *p* = .004); refer to Fig. 3–5. There were no differences by condition in change in hopelessness (*p*s > .08) or depression change expectancies (*p*s > .05).

None of the SSIs reduced or increased mental health outcomes at four-week follow-up more or less than the active comparator *Behavioral Activation* SSI (*p*s > .06; see Secondary Analysis H3 in the Supplement). Secondary analyses compared the average change in outcomes between the control condition and the mean of all SSIs. From baseline to post-test, SSIs outperformed the control on every outcome (*d*s: 0.12 – 0.21; *p*s 2.47*10^−7^ – 2.08*10^−20^); at week four, SSIs showed greater reductions in depression (*d* = 0.07, *p* = .04) and greater increases in agency (*d* = 0.08; *p* = .001), but smaller increases in readiness to change (*d* = −0.05, *p* = .045), while hopelessness (*p* =.67) and depression change expectancies (*p* =.15) did not differ (see Secondary Analysis H6 in the Supplement). Sensitivity analyses that added covariates to the depression analysis produced substantively similar results to the primary model (See Sensitivity Analyses in the Supplement).

SSIs’ effects on outcomes at post-test were correlated with their effects at week four in agency (*r*(10) = 0.66, *p* = .02), but not hopelessness (*r*(10) = 0.34, *p* = .28), depression change expectancy (*r*(10) = 0.36, *p* = .25), or readiness for change (*r*(10) = 0.55, *p* = .06; refer to Correlation Between Post-test and Week Four Effects in the Supplement).

#### Robustness Checks

When adjusting for multiple comparisons between SSIs and the control, most of the differences at post-test remained statistically significant, but all differences between conditions at four-week follow-up were no longer statistically significant, except the greater rise in agency in *Interactive Cognitive Reappraisal* (*adjusted p* = .001), and the lower rate of aha! moments in *Inner Child Healing Walk* (*adjusted p* = .02). Re-running the analyses with either the complete sample (i.e., including participants who were likely randomized multiple times) or the most exclusive sample (i.e., removing all responses with a repeated IP address at baseline) produced similar results to the original models. Analyses accounting for differential attrition across conditions using inverse probability weighting also produced similar results to the original models. Thus, our findings are robust to varying analytic approaches. Refer to Robustness Check in the Supplement.

### SSI Acceptability and Expected Benefit

Fig. 5 shows participant ratings of SSIs’ acceptability and expected benefit at post-test. Refer to Secondary Analysis H4 in the Supplement for acceptability comparisons between the active comparison SSI (*Behavioral Activation*) and all other SSIs.

### Moderators of Change

A pre-registered analysis showed participants who expected they could improve their depression more at baseline had greater decreases in depression from baseline to week four (*b* = −0.08, *t*[6707] = −2.85, *p* =.004). An exploratory analysis limited to participants who were assigned to an SSI found those who hadn’t accessed any form of formal mental health support in the past month had a significantly greater decrease in depression from baseline to week four (*b* = −0.62; *t*[5327] = −4.02; *d* = −0.12; 95% CI −0.06, −0.18; *p* = 5.82*10^−5^).

Points reflect model estimates of a condition’s average change from baseline to four-week follow-up compared to the control condition. Error bars reflect 95% confidence intervals; non-overlapping confidence intervals are not evidence of statistically significant differences between SSIs. Blue reflects a difference from the control in the desired direction, and red reflects the opposite. Grey bars reflect statistically insignificant differences from the control. * *p* < 0.05, ** p<0.01, *** p<0.001

Points reflect model estimates of a condition’s average change from baseline to four-week follow-up compared to the control condition. Error bars reflect 95% confidence intervals; non-overlapping confidence intervals are not evidence of statistically significant differences between SSIs. Blue reflects a difference from the control in the desired direction, and red reflects the opposite. Grey bars reflect statistically insignificant differences from the control. * *p* < 0.05, ** p<0.01, *** p<0.001

Values reflect Cohen’s *d* effect sizes expressing the difference in the change from baseline to the stated timepoint compared to the control condition. Blue reflects change in the desired direction and red reflects the opposite. Hopelessness and depression were reversed to standardize effect direction.

Points reflect the average rating on each outcome, except for those in the top right plot, which refer to the percentage of participants who experienced an aha! moment. Error bars reflect 95% confidence intervals. Shaded regions reflect the error bar of the behavioral activation SSI. Non-overlapping error bars differ significantly (*p* < .05).

## Discussion

SSIs are beginning to fill crucial gaps in care, enabling more people to access reliable support in moments of need (Schleider & Beidas, 2022). Learning about the strengths and weaknesses of various kinds of SSIs can help to make the next generation of SSIs more effective and appealing. We crowdsourced 66 diverse SSIs for depression from global teams and conducted an experiment (*N* = 7,505) to evaluate the 11 most promising ones against an evidence-based active comparator and an attention control.

Many SSIs improved depression-relevant outcomes in the immediate term (*d*s≤ 0.37), but these effects had substantially weakened four weeks later (*d*s≤ 0.16). At four-week follow-up, only two of the SSIs (*Interactive Cognitive Reappraisal* [*d* = 0.15]and *Mindful Attention Skills* [*d* = 0.14]) statistically significantly alleviated depression, though others had effect sizes that were nearly as large. Results highlight the efficacy and appeal of several kinds of SSIs, some reflecting current design practices and others introducing novel approaches. For example, many of the video-based SSIs – a format that had not been previously studied – were efficacious and well-liked. The most successful SSIs at post-test and four-week follow-up prioritized clear, solution-focused guidance for facing depression. The least successful did not address a key depression-relevant problem for users and failed to focus on a memorable message.

### Interpreting Results

The SSI that most reduced participants’ PHQ-9 (depression) scores at four-week follow-up (*Interactive Cognitive Reappraisal*) did so by 0.76 points (*d* = 0.15) more on average than the control. When effects were averaged across all 12 SSIs, the mean reduction was only slightly greater than the control’s (d = 0.07). While these effects are smaller than those of more intensive psychological interventions, they may still feel worthwhile to many, especially when SSIs take only 10 (often enjoyable) minutes. As noted, because SSIs are low-cost, highly scalable, and minimally burdensome, these small individual effects could add up to a meaningful public health impact.

#### Potential Iatrogenic Effects on Longer-term Readiness to Change

Surprisingly, at four-week follow-up, participants assigned to many of the SSIs appeared to express slightly lower readiness to make changes toward overcoming depression than those assigned to the control (though only statistically significantly so for one SSI, *Personalized Intervention Recommender*). It could be that once the initial boosts in confidence and motivation following an SSI faded, participants felt let down because they got their hopes up for a change that never came. If this interpretation is correct, it may represent the first evidence of an iatrogenic effect in SSIs.

Alternatively, this finding might reflect an issue with participants’ interpretation of the items on the readiness to change measure. Specifically, participants in the control condition may have expressed higher readiness to change because they had only received information about trout so far, and remained *ready to receive an intervention* because the study was advertised as “Help test an online mental health resource.” Another possibility is that people who completed an SSI were not interested in change because they already experienced benefits from the SSI. Further research should explore if this reflects a lasting iatrogenic effect and if it is unique to SSIs or common to other psychological interventions.

#### Why Some SSIs May Have Outperformed Others

Because the experiment lacked statistical power for high-resolution comparisons between SSIs, efforts to identify why some SSIs outperformed others are inherently tentative. However, in broad strokes, our findings suggest that to encourage hope and agency toward future improvement, SSIs must provide clear and actionable guidance that directly addresses an important problem for users. The most efficacious SSIs, such as *Interactive Cognitive Reappraisal* and *Mindful Attention Skills*, focused on a single practical skill or behavior with a compelling call to action and an emphasis on helping users apply learnings into their daily lives.

In contrast, SSIs like *Inner Child Healing Walk* and *Personalized Intervention Recommender* may have felt too abstract because they were not solution-focused. Similarly, although *Savoring Strategies* encouraged real-world practice, the savoring skills it introduced may not have resonated with participants because they did not directly address depressive symptoms. And while *5 Habits to Beat Depression* was well-liked for its engaging and credible presentation, its rapid presentation of five habits might have left participants feeling they lacked a clear path to change.

The SSIs users found most compelling (in terms of credibility and expected benefit as well as “aha!” moments) were not necessarily those with the highest-quality presentation. Several visually and functionally simple SSIs were rated as highly acceptable. Meanwhile, the SSI with the lowest credibility and expected benefit score, *Inner Child Healing Walk*, was one of the most visually and conceptually rich. Overall, while a compelling presentation can enhance an SSI’s appeal, providing useful intervention content is essential.

Some SSIs, such as *Interactive Cognitive Reappraisal* and *Mindful Attention Skills*, were efficacious and appealing across outcomes and measurement timepoints. Both SSIs focused on a single evidence-based skill and emphasized how users could apply it to their lives. Yet they also differed in key ways. *Interactive Cognitive Reappraisal* was text-based, while *Mindful Attention Skills* centered on cinematic video lessons.

Notably, *Mindful Attention Skills’* key message – that training attention gives greater agency over negative thoughts – was similar to that of *Reframing Negative Thoughts*, the SSI with the weakest effect on depression at four weeks. However, the SSIs differed in several ways. For one, *Mindful Attention Skills* featured engaging videos, while *Reframing Negative Thoughts* was purely audio. *Mindful Attention Skills* also included components intended to normalize users’ struggles, presented a memorable story emphasizing its central argument, and encouraged regular practice via interactive exercises, while *Reframing Negative Thoughts* lacked interactive exercises altogether.

While the SSIs’ effects on outcomes at post-test correlated fairly well with their effects at week four, there were some notable cases when an SSI performed especially well on an outcome at post-test but especially poorly at week four, and vice-versa. For example, *Reframing Negative Thoughts* decreased hopelessness more than any other SSI at post-test, but had nearly the weakest effect on it at week four. This may suggest some SSIs are more effective in the short-term than they are in the long-term; for example, perhaps *Reframing Negative Thoughts’* lack of interactive exercises applying concepts to users’ daily lives held back its long-term impact. Alternatively, this variation might again reflect statistical noise arising from insufficient power to compare effects across SSIs and a lack of correction for multiple comparisons.

Our conclusions about the elements that made some SSIs more successful than others align with existing SSI design norms, such as the B.E.S.T.T. elements of SSI design (Manvelian et al., 2025; Schleider, Dobias, Sung, Mumper, et al., 2020). Yet, they also highlight the value of novel approaches, like using video as a content delivery medium. Ultimately, SSIs’ effectiveness owes to a complex interplay of factors, including match to the target population’s preferences, delivery format, emotional resonance, and user burden. Other important design factors may be difficult to operationalize – both of the leading SSIs in this study, *Interactive Cognitive Reappraisal* and *Mindful Attention Skills*, are the products of nearly 10 years of iterative design that likely amount to more than the sum of their intervention components (Morris et al., 2015, Mrazek et al., 2022).

### Strengths and Limitations of the Study

This study had several strengths, including being the largest trial of SSIs to date, having low attrition, using a large and diverse sample achieved via broad entry criteria and nationwide recruitment, and including two comparator conditions: a control and an active comparison SSI. In addition, the SSIs included for testing reflected a global crowdsourcing effort and a rigorous selection process.

Our choice to conduct our study using the online recruitment platforms CloudResearch Connect and Prolific had several advantages. Recruiting such a large sample on social media would have been difficult given the problem of fraudulent research participants (Kumarasamy et al., 2024). The rates of attrition, within and between sessions, on the platforms we used are much lower than typical for social media recruiting, which alleviated differential dropout (Chapkovski et al., 2024). However, this choice of sample also had weaknesses. First, participants were paid online workers, who may differ from the broader populations SSIs aim to serve (Protzko, 2022). For example, many participants were likely not actively seeking mental health support when they found the study, so we did not reach them at moments of need when SSIs might be more helpful. Overall, caution is needed when generalizing our results beyond the population sampled: nonprobability opt-in panels of adult online workers experiencing elevated (mostly moderately elevated) depressive symptoms. Again, data from a variety of settings outside of clinical trials are needed.

This being the first megastudy of mental health interventions, we aimed to include a wide range of intervention strategies and styles. This choice had both strengths and limitations. One strength was that it made the study more reflective of all possible designs and increased our odds of finding unexpectedly fruitful SSIs. Yet, this variety also made it harder to know *why* an SSI succeeded. With SSIs differing on several features, we could not be confident that a given feature led to greater efficacy.

To further expand the range of SSIs in the study, we took efforts to solicit SSI submissions from diverse teams, including people with no involvement in research on SSIs or mental health. Yet, the majority of submissions we received came from people connected to academic research, likely because we shared the project via our core team’s online social networks (i.e., mostly researchers), the fact that most people without a particular interest in mental health research likely do not have strong ideas about SSI design. More extensive and targeted advertising to communities outside of mental health research, as well as monetary prizes for successful submissions, may have helped to expand the diversity of submitters’ backgrounds and perspectives.

Another source of strengths and weaknesses was the study’s statistical power. On one hand, the study had by far the largest sample of any study of SSIs. On the other hand, most of the comparisons in the study were still not sufficiently powered to detect effects that might be valuable. A useful target would be the average effect size of SSIs for youth depression observed in Ball et al.’s (2024) meta-analysis, *g* = −0.12. Smaller effects would be helpful to compare effects across SSIs, though detecting such effects would require many more participants per condition than we had in this study.

### Future Directions

One avenue for future work is to study the SSIs that showed the greatest promise in this megastudy in greater depth; while this study showed *which* SSIs are efficacious, future work can more closely examine *why*. For example, future megastudies might hold elements like style or intervention content constant across SSIs to pinpoint active elements and mechanisms of change. Alternatively, future work might continue our exploratory approach by examining SSI designs that are entirely distinct from those we tested. A related direction is to apply our findings to psychosocial interventions more broadly. SSIs or elements of SSIs identified as efficacious in this study might be combined or integrated into longer behavioral interventions.

#### Implementation

A crucial future direction is implementing SSIs into systems that can maximize their impact. Even if SSIs are effective and appealing, simply making them freely available on a public website is unlikely to spark organic virality in today’s competitive attention economy. Thoughtful implementation into settings with wide reach, like social media and schools, or high need, like therapy waitlists, is key.

Despite their successes, existing SSIs have not achieved digital mental health’s holy grail: organic uptake by the millions of people underserved by current mental health solutions. This lack of reach might reflect myriad barriers, including low public awareness of SSIs and the pathways to accessing them. If SSIs can be successfully implemented on a large scale, effect sizes as small as those we saw at four-week follow-up could still make a substantial public health impact (Dodge et al., 2024). Massive reach is not the only pathway to impact, but it is a particularly important one for digital, self-guided SSIs given their small average effects.

SSIs’ appeal is an essential component of their path to impact. While this study’s measures are a useful signal as to the kinds of SSIs that users resonate with, real-world data on appeal is much more valuable. For example, the motivational video central to the *Moral Elevation* SSI achieved virality, garnering over 120 million views on YouTube since 2014 (https://www.youtube.com/watch?v=uaWA2GbcnJU). While Moral Elevation was not the most efficacious SSI in our megastudy, it may still have the greatest impact.

Large-scale implementation also requires careful attention to potential negatives. Expanding the populations an SSI reaches might change its average effects and lead to unwanted spillover effects, as in the possible drop in long-term readiness to change we observed in this study (Bryan et al., 2021; Nafziger, 2020). Similarly, SSIs’ messaging might be misinterpreted by some, especially because self-guided SSIs lack monitoring and opportunities for clarification. For example, some users might misinterpret an SSI that encourages facing challenging situations as suggesting they pursue a dangerous activity. Such adverse events require intentional tracking with harmonized assessments beyond average change in mental health outcomes (Arnautovska & Milton, 2025).

#### Leveraging Short-term Gains

Low hope and agency often prevent people experiencing depression from seeking care or taking other positive action (Garrey et al., 2022; McDermott et al., 2017). Our short-term results make it clear that many users finish SSIs with a sense that further improvement is possible, especially when SSIs provide intervention content that users can apply to their lives. However, by four-week follow-up, these effects had washed out, and readiness to change was lower among those assigned to an SSI than the control.

One way to boost SSIs’ efficacy may be to leverage these short-term gains in agency and hope for improvement to encourage longer-term engagement in behavior change and provide opportunities for service uptake. For example, SSIs could be used as entry points to a stepped-care model, in which people who complete an SSI are referred to longer-term digital health tools (Rizvi & Kleinman, 2023, Wakefield et al., 2021). Other work might help users to find the right SSI for them, using machine learning treatment-matching models or simply letting users choose from a variety of SSIs.

## Conclusions

We gained new insights into the efficacy of digital, self-guided SSIs to reduce depression. Our findings suggest that several kinds of SSIs can be useful in the short term, but that some are more promising than others. SSIs that introduced an evidence-based skill or behavior with a presentation that was focused, actionable, and directly relevant to users’ needs seemed to perform especially well. Work evaluating SSIs outside of paid study contexts is needed to determine real-world effectiveness.

## Methods

### Part 1: Crowdsourcing Interventions

#### Overview:

We advertised an open call for SSIs for depression and received 66 submissions. The 50 submitting teams were based in five continents and involved 112 contributors, including students, popular YouTubers, and representatives of leading mental health apps. Next, a team of digital mental health researchers and people with lived experience of depression chose a group of SSIs reflecting a range of promising approaches for further testing.

##### Crowdsourcing Intervention Submissions

We identified SSIs for the megastudy via crowdsourcing, with an open call for submissions from March to May 2024. Our crowdsourcing process largely followed the example set by a previous crowdsourced megastudy: the Strengthening Democracy Challenge (Voelkel et al., 2024).

We created a website, handbook, and video explaining the project and how to submit. Next, members of our study team posted about the megastudy on social media and online communities on Reddit and Discord, aiming to reach as many people as possible with a broad range of backgrounds and perspectives. We also invited several content creators (e.g., YouTubers) who we believed had relevant content to submit.

Anyone 13 years or older could submit to the megastudy. One could submit either an SSI or a description of a SSI (provided our teams could feasibly create the SSI together within the next few months). Any kind of SSI was eligible for review if it was under 10 minutes, not hateful or disturbing, online, scalable, self-guided, costless, and comprehensible to English speakers. The submission portal asked about the submitting team’s background, the SSI’s theory of change, and how the SSI might be disseminated; all of these questions are listed athttps://osf.io/8gys7. Submitters were not compensated. However, we stated that if an SSI was selected for testing, all members of the submitting team would be offered authorship on this paper.

##### Choosing Submitted Interventions to Include in the Megastudy Experiment

Before crowdsourcing SSIs, we decided to include one *active comparison* SSI in the experiment: an evidence-based SSI for depression against which to compare the submitted SSIs. For this purpose, we used the adult version of the Action Brings Change Project (referred to here as *Behavioral Activation*), an SSI originally developed for teens that has shown efficacy in multiple trials with youth samples (Schleider et al., 2022; Schleider, Mullarkey, et al., 2019).

We received 66 SSI submissions and planned to include 10 for testing. To decide which of the submitted SSIs to include, we used a six-stage evaluation process involving teams of reviewers with varying areas of expertise, as well as 335 pilot testers (refer to https://osf.io/z43nq for information on this evaluation process). Broadly, our selection process balanced two motivations: to identify SSIs with the greatest potential for real-world effectiveness, and to include a diverse range of SSI designs. Thus, we had to consider trade-offs such as evidence vs. novelty, as well as efficacy vs. broad appeal.

After selecting nine SSIs, the editorial board decided that the submitted SSIs did not reflect a sufficiently broad range of intervention approaches. As a result, the board decided to invite an additional SSI submission from a team known to have created evidence-based dialectical behavioral therapy-centered digital content. That team submitted an SSI (a subset of *Dialectical Behavioral Therapy Skills*), and the editorial board decided to include it for testing. Four months later, the editorial board decided to include a 12th SSI (*Reframing Negative Thoughts*) from *Calm Health* as thanks for making a monetary donation to the organizing team (led by JLS) that enabled data collection for this study. The *Calm Health* team had no input in analysis or manuscript writing beyond authorship (CJM and KSH). None of the SSIs tested came from core authorship team members’ own teams, though several of the included SSIs’ submitting teams had collaborated with members of the core authorship team in the past.

##### Interventions

The 12 SSIs varied in format (e.g., video vs. text), intervention approach (e.g., cognitive behavioral therapy vs mindfulness practice), and their creators’ backgrounds (e.g., clinical researchers vs. popular online content creators). The *attention control* condition aimed to require as much attention as the other SSIs without influencing depressive symptoms or mood (a three-minute video, multiple-choice questions, reading passage, and writing exercise, all about trout). Refer to Table 1 for information on conditions.

Materials for these conditions, including links to their browser versions, are online at https://osf.io/agvh6

Three raters (AS, EKS, BK) rated each SSI on various characteristics, then met to come to an agreement on these ratings. The characteristics were *Video-based, Engaging, Interactive, Personalized, Effortful, Refers to outside mental health resources, Contains meditation exercise, Contains features of cognitive reframing, Contains features of behavioral activation, Contains features of mindfulness, Created by a team led by clinicians or clinically-trained mental health researchers, and Provides a take-home reminder*. These ratings are provided at https://osf.io/qbpk6.

Among the 11 teams whose SSIs were tested, nine included at least one member who was a digital mental health researcher, eight included a clinical psychologist, four included a researcher from a field that was not psychology, three included members representing mental health companies, and three included undergraduate students.

### Part 2: Megastudy Experiment

#### Overview:

Participants (target *N* = 7,500) were randomized to one of 13 conditions to evaluate SSIs’ efficacy in improving depression and related outcomes over four weeks.

##### Participants

Participants were recruited from the online participant recruitment platforms CloudResearch Connect and Prolific (Hartman et al., 2023; Palan & Schitter, 2018). Participants were invited to participate in study sessions and compensated through their online recruitment platform. All other study activities took place through the online survey and experience management platform Qualtrics, via users’ personal computers, tablets, or smartphones (*Qualtrics*, 2005). Participant randomization was automated through Qualtrics as well.

#### Inclusion criteria.

All inclusion criteria were pre-registered before data collection. Participants needed to be located in the United States, at least 18 years old, and able to read and write fluently in English. They also had to score at least 10 on the PHQ-9 screen during the screen survey, suggesting moderate depression (Kroenke et al., 2001). We included all participants who were randomized to a condition (and thus completed baseline measures) in the analyses (intention-to-treat). If a participant began a survey multiple times, we only included data from the first session in analyses.

##### Procedure

First, participants completed a screen survey where they responded to the Patient Health Questionnaire (9-item, PHQ-9) and encountered a “honeypot” item checking for bots (i.e., an element of the survey that humans shouldn’t usually find; https://osf.io/kba7v). Participants who failed the honeypot were automatically prevented from continuing in the study. Eligible participants were then asked to consent to participate in the study. After consenting, participants immediately began the baseline well-being questionnaire. Next, they were randomly allocated to one of the 12 SSIs or a control condition. After completing the treatment, they were asked to respond to some of the baseline measures again, as well as measures of satisfaction with the SSI. Four weeks later, participants were invited to a follow-up survey with the baseline well-being measures again and several SSI satisfaction measures.

Participants were compensated after completing each study session: $0.25 for completing the eligibility screen (1 minute), $3.00 for completing the first study session (18 minutes), and $1.00 for completing the four-week follow-up (5 minutes). Participants who were randomized to a condition were invited to complete the follow-up. After each study session, participants were offered a list of online mental health resources (refer to https://osf.io/wz5u6). At the end of the follow-up survey, participants were given access to online versions of all of the SSIs in the study (refer to https://osf.io/nqdte).

##### Measures

We evaluated outcomes using self-report measures. Refer to Table 2 for measure collection timepoints, and https://osf.io/76cfy for all items. For each participant, at each measurement timepoint, we randomized the order in which measures were presented.

To measure *depressive symptoms*, we used the nine-item Patient Health Questionnaire (Kroenke et al., 2001). In this scale, participants rate how often they have been bothered by nine concerns over the past two weeks (e.g., “Poor appetite or overeating”) on a scale from 0 (“Not at all”) to 3 (“Nearly every day”). The total score ranges 0–27, with higher scores indicating more severe depression. The PHQ-9 had a Cronbach’s α of 0.75 at baseline, suggesting it was an internally consistent measure of depressive symptoms in this sample and session.

We measured participants’ *positive actions and thoughts* (e.g., engaging in social behaviors and challenging their negative thoughts) using the Frequency of Actions and Thoughts Scale (FATS, Terides et al., 2016). The scale includes 12 items about one’s experiences over the past week. Items are rated from 0 (“Not at all”) to 4 (“Every day”), with total scores ranging 0–48. The FATS had α = 0.76 at baseline.

To measure *agency*, we used the Pathways Subscale of the State Hope Scale, a three-item self-report measure of one’s perceived ability to generate plans and work toward goals (Snyder et al., 1996). The scale asks about respondents’ beliefs “here and now.” Items are rated from 1 (“Definitely false”) to 8 (“Definitely true”), with total scores ranging 3–24. The measure had α = 0.85 at baseline.

To measure *hopelessness*, we used the four-item Beck Hopelessness Scale (Perczel Forintos et al., 2013). The scale asks whether various statements are typical, with no specific time frame. Items are rated from 1 (“Rarely typical”) to 3 (“Very typical”), with total scores ranging 4–12. The BHS-4 had α = 0.87 at baseline.

We evaluated readiness to make changes toward overcoming depression using two multiple-choice questions rated from 1 (“Not at all”) to 4 (“Extremely”): “How important is making changes toward overcoming depression to you right now?” and “How confident are you about making changes toward overcoming depression?”. We summed these items into a readiness to change score ranging from 2–8. Readiness to change had α = 0.46 at baseline.

We measured how much participants expected they would be able to change their feelings of depression using the three items from the Depression Change Expectancies Scale (DCES) with the highest item-total correlations in the original study on the scale (Eddington et al., 2014). Items were rated from 1 (“Strongly disagree”) to 5 (“Strongly agree”), with a total score ranging 3–15. The DCES had α = 0.75 at baseline.

We measured how acceptable participants found their intervention with a few measures. First, the Credibility / Expectancy Questionnaire (CEQ), with scores ranging from 4 to 56 (Devilly & Borkovec, 2000). Some of the items are rated 1 (“Not at all”) to 9 (“Very much”), and others are rated 0 (0%) to 10 (100%). The CEQ had α = 0.92 at baseline.

We collected the following demographic information about participants: age, gender, race, ethnicity, education, household income, relationship/marital status, political party, employment status, disability or chronic health condition, socioeconomic status (MacArthur Social Ladder, ordinal 1–10; Adler et al., 2000), self-identification as having depression (yes/no), number of years living with depression (continuous), and having accessed various forms of mental health support (yes/no for each form of support). All variables mentioned in this paper were self-reported by participants. Some categorical variables’ levels were determined by the researchers (e.g., disability or chronic health condition), and others by CloudResearch Connect (e.g., gender and race).

##### Primary Hypothesis

For each SSI, we hypothesized that participants assigned to the SSI would report a different extent of change in depressive symptoms between baseline and four-week follow-up than participants assigned to the control condition.

##### Primary Analysis

We used a mixed-effects linear regression model to test if depressive symptoms changed to a different extent from baseline to four-week follow-up in each of the 12 SSIs compared to the control condition. Timepoint is a two-level factor (baseline and week four follow-up). The model included a participant identifier as a random intercept:

$${\text{Y}}_{\text{ij}}=\beta_{0}+\beta_{1}\cdot {\text{timepoint}}_{\text{ij}}+\beta_{2}\cdot {\text{condition}}_{\text{ij}}+\beta_{\text{3}}\cdot \left({\text{timepoint}}_{\text{ij}}\times {\text{condition}}_{\text{ij}}\right)+{\text{u}}_{\text{i}}+\varepsilon_{\text{ij}}$$

##### Secondary Hypotheses and Analyses, Sensitivity Analyses, and Robustness Checks

We pre-registered six secondary hypotheses examining SSIs’ effects on secondary outcomes, comparisons between SSIs, and moderators of treatment effects. We also pre-registered three sensitivity analyses examining the effects of covariates and differential attrition, as well as a robustness check examining the effects of multiple comparisons. The results of each are presented in the Supplement.

##### Inference Criteria

We used *p*-values as our criterion for statistical significance, pre-registering that we would report effects of *p* ≤ .05 as significant, .05 < *p* ≤ .1 as “marginally significant,” and *p* > .1 as nonsignificant. However, for simplicity’s sake, we decided to eliminate the “marginally significant” category in this paper and simply report p < .05 as significant and all else as nonsignificant.

We used two-sided tests for all analyses. Unless otherwise indicated, results that mention differences (e.g., greater, weaker) reflect statistically significant differences. To obtain Cohen’s d estimates from a binary predictor in a mixed effects model, we divided the predictor’s regression coefficient by its standard deviation at baseline, computed as the outcome’s between-person variance (random intercepts) plus its within-person (residual) variance (Feingold, 2009).

We ran mixed-effects regression models using the *lme4* and *lmerTest* packages in R version 4.4.2 (Bates et al., 2015; Kuznetsova et al., 2017; R Core Team, 2015). We visualized analyses using the *sjPlot* and *ggPlot2* R packages (Lüdecke et al., 2021; Wickham et al., 2019).

##### Target Sample Size Calculation

We aimed to recruit 500 participants per intervention condition and 1,500 for the control condition (because tripling the sample size in the control improved the power we could generate from the same number of participants in analyses comparing the intervention conditions to the control). Thus, we planned to recruit 7,500 participants in all.

We pre-registered this sample size to detect change from baseline to four-week follow-up between each intervention and the control condition with an effect size of *d* = 0.17 or larger (i.e., roughly a 0.78-point difference on the PHQ-9 score) with 90% power and alpha = 0.05. This sample size also enabled the detection of differences in change from baseline to follow-up between each intervention condition of *d* = 0.19 or larger (i.e., roughly a 0.88-point difference on the PHQ-9 score). Based on estimates from previous research, power analyses assumed a test-retest correlation of *r* = 0.68 for PHQ-9 score over four weeks (Sun et al., 2020), and that 20% of participants who completed the baseline would not complete the four-week follow-up (Kaveladze et al., 2025).

##### Open Science

We pre-registered the procedures, hypotheses, analysis plan, measures, and decision criteria before collecting data (available online at https://cdn.clinicaltrials.gov/large-docs/68/NCT06856668/Prot_SAP_ICF_001.pdf). The analysis scripts, study materials, analyzed datasets (after publication), and SSIs from this study are publicly available at https://osf.io/agvh6.

## Supplementary Material

Supplementary Files

This is a list of supplementary files associated with this preprint. Click to download.

- megastudyanalysisnotebook.pdf
- megastudydataandscriptsdeidentifiedtoshare.zip

## Figures and Tables

**Figure 1:**
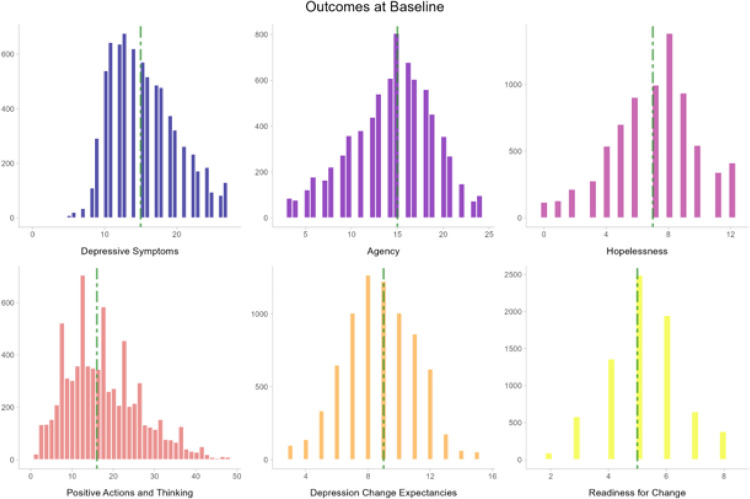
Distributions of Mental Health Outcomes at Baseline in a Megastudy of 12 Single-Session Interventions for Depression (*N* = 7,505)

**Table 2.** Experimental Conditions in a Megastudy (*N* = 7,505) of Adult Online Workers Experiencing Depressive Symptoms

Condition	Description	Prior Testing
5 Habits to Beat Depression	A clinical psychologist and science communicator describes five one-minute habits to beat depression.	None
AI-assisted Personalized Narrative	Users describe a negative thought they struggle with and a large language model system generates a story in which someone overcomes that thought. Users are prompted to reflect on their feelings and the extent to which they believe each thought.	RCT (Bhattacharjee et al., 2025)
Dialectical Behavioral Therapy Skills	A series of videos that present a subset of skills from dialectical behavioral therapy, aiming to regulate and manage negative emotions.	RCT of longer format (Rizvi et al., 2022)
Interactive Cognitive Reappraisal	An interactive and aesthetically pleasing text-based SSI in which participants learn the value of reframing negative thoughts, then reframe a thought of their own.	RCT of longer format (Morris et al., 2015)
Inner Child Healing Walk	An interactive animated journey to reconnect and heal one’s inner child through breathing exercises, positive affirmations, and supportive animal companions.	None
Mindful Acceptance	A clinical psychologist explains ways to use mindful acceptance to deal with difficult thoughts and feelings.	RCT of longer format, unpublished (https://drive.google.com/file/d/1mwVIe20JMO10abDiRWUK_WovphurxJA/view)
Mindful Attention Skills	A series of videos explain how to identify counterproductive evaluations and replace them with new evaluations that feel both true and helpful.	RCT of longer format (Mrazek et al., 2022)
Moral Elevation	Users learn about moral elevation, then watch a touching video (a Thai life insurance advertisement with over 120 million views as of July 2025) where a man does good deeds for people in his community. Finally, they plan an action to help others in their own life.	RCT of longer format (McGuire et al., 2023)
Personalized Intervention Recommender	An interactive SSI that identifies the depression symptom one would most like to improve and offers a personalized recommendation for support options (e.g., a self-guided mental health app or a professional therapist), matching one’s preferences and capacity.	None
Reframing Negative Thoughts	An audio-only program in which users are guided to reflect on how they might challenge negative automatic thoughts and beliefs they struggle with.	RCT of longer format (Huberty et al., 2019)
Savoring Strategies	An interactive SSI targeting mood. It introduces strategies to savor positive memories, the present moment, and future events.	None
Behavioral Activation (Active Comparator SSI)	An interactive behavioral activation SSI where users create an “action plan” of positive social and personal actions that can improve their well-being. This SSI has demonstrated efficacy and reflects current “best practices” in SSI design.	RCTs (Schleider et al., 2022; Schleider, Mullarkey, et al., 2019)
All About Trout (Attention Control)	An educational program with information and interactive exercises about trout fish. Intended to require some attention and effort but minimally influence depressive symptoms and mood.	None

**Table 3.** All Primary, Secondary, and Screening Outcome Variables Collected During a Megastudy (N = 7,505) Comparing 12 SSIs for Depression, by Timepoint Administered

Measure	Example item	Screen	Baseline	Post-test	4-week follow-up
Patient Health Questionnaire - 9 (primary)	How often have you experienced little interest or pleasure in doing things?	X	X		X
Frequency of actions and thoughts scale (secondary)	How often did you change your thinking to be more realistic and helpful?		X		X
Agency: pathways subscale of the state hope scale (secondary)	There are lots of ways around any problem that I am facing now.		X	X	X
Beck Hopelessness Scale - 4-item (secondary)	I feel that the future is hopeless and that things cannot improve.		X	X	X
Readiness to Change (Secondary)	How confident are you about making changes toward overcoming depression?		X	X	X
Depression Expectancies for Change - 3 item version (secondary)	If I work hard, I can have a positive impact on my problems with depression.		X	X	X
Credibility and Expectancy Questionnaire (secondary)	How confident would you be in recommending this program to a friend who experiences similar problems?			X	
